# Inhibition of Calcium Influx Reduces Dysfunction and Apoptosis in Lipotoxic Pancreatic β-Cells via Regulation of Endoplasmic Reticulum Stress

**DOI:** 10.1371/journal.pone.0132411

**Published:** 2015-07-06

**Authors:** Yuren Zhou, Peng Sun, Ting Wang, Kaixian Chen, Weiliang Zhu, Heyao Wang

**Affiliations:** 1 Shanghai Institute of Materia Medica, Chinese Academy of Sciences, 555 Zuchongzhi Road, Shanghai, 201203, China; 2 School of Pharmacy, Shanghai University of Traditional Chinese Medicine, 1200 Cailun Road, Shanghai, 201203, China; Niigata University Graduate School of Medical and Dental Sciences, JAPAN

## Abstract

Lipotoxicity plays an important role in pancreatic β-cell failure during the development of type 2 diabetes. Prolonged exposure of β-cells to elevated free fatty acids level could cause deterioration of β-cell function and induce cell apoptosis. Therefore, inhibition of fatty acids-induced β-cell dysfunction and apoptosis might provide benefit for the therapy of type 2 diabetes. The present study examined whether regulation of fatty acids-triggered calcium influx could protect pancreatic β-cells from lipotoxicity. Two small molecule compounds, L-type calcium channel blocker nifedipine and potassium channel activator diazoxide were used to inhibit palmitic acid-induced calcium influx. And whether the compounds could reduce palmitic acid-induced β-cell failure and the underlying mechanism were also investigated. It was found that both nifedipine and diazoxide protected MIN6 pancreatic β-cells and primary cultured murine islets from palmitic acid-induced apoptosis. Meanwhile, the impaired insulin secretion was also recovered to varying degrees by these two compounds. Our results verified that nifedipine and diazoxide could reduce palmitic acid-induced endoplasmic reticulum stress to generate protective effects on pancreatic β-cells. More importantly, it suggested that regulation of calcium influx by small molecule compounds might provide benefits for the prevention and therapy of type 2 diabetes.

## Introduction

During the development of type 2 diabetes (T2D), obesity induced elevation level of free fatty acids (FFAs) causes both insulin resistance and pancreatic β-cell failure [1, 2]. And early appearance of β-cell failure could subsequently lead to insufficient insulin secretion, thereby breaking normal glycemic control [1]. It is known that FFAs play an important role in the normal function of pancreatic β-cells. However, pleiotropic effects of FFAs have also been verified [3]. FFAs supply could augment glucose-stimulated insulin secretion, while chronically in excess, FFAs can impair insulin biosynthesis, secretion and induce β-cell apoptosis [2, 3]. Nonetheless, the molecular mechanisms of FFAs-induced β-cell failure are complex and not fully understood.

Under physiological conditions, acute stimulation of FFAs could activate receptors in pancreatic β-cells, such as G-protein coupled receptor 40 (GPR40), to amplify insulin secretion pathway via increasing intracellular calcium concentration [4, 5]. Medium- and long-chain FFAs like palmitic acid (PA) could stimulate voltage-sensitive Ca^2+^ influx and directly mobilize Ca^2+^ from intracellular endoplasmic reticulum (ER) Ca^2+^ pools in pancreatic β-cells [6, 7]. Therefore, chronic elevate FFAs could persistently augment Ca^2+^ metabolism in mitochondria, which might be related to cell apoptosis [8]. More importantly, sustained elevation of intracellular Ca^2+^ concentration ([Ca^2+^]_i_) could induce ER-stress response, as β-cells have a well-developed ER and are highly susceptible to ER-stress [9, 10]. Together factors indicate that Ca^2+^ signal is strongly involved in FFAs-induced β-cell dysfunction and apoptosis. It has been reported that some Ca^2+^ chelators or Ca^2+^ signal blockers had a protective effect on FFAs-induced β-cell apoptosis [11, 12]. Meanwhile, our previous study revealed that using a small molecule antagonist of GPR40 to block Ca^2+^ release also reduced PA-induced apoptosis in pancreatic β-cells [13]. Thus, regulation of Ca^2+^ release might provide benefit for β-cell protection during the development of T2D.

The aim of this study was to investigate the possible effect of inhibition of sustained Ca^2+^ influx on lipotoxic β-cells. Using an classic L-type Ca^2+^ channel blocker nifedipine, which has been reported to inhibit Ca^2+^ influx and mediate insulin secretion in pancreatic β-cells [14] and diazoxide, a potassium channel activator which could also block Ca^2+^ influx during GSIS [15], the effects of regulation of Ca^2+^ influx on chronic PA-treated pancreatic β-cells were studied.

## Materials and Methods

### Cell culture and murine pancreatic islets isolation

Mouse insulinoma cell line MIN6 cells were kindly provided by Prof. S. Seino [16]. The cells were cultured in Dulbecco’s modified Eagle’s medium (DMEM) supplemented with 10% fetal bovine serum (FBS), 25 mM glucose and 50 μM β-mercaptoethanol at 37°C under 5% CO_2_. All cell culture reagents were purchased from GIBCO (Carlsbad, CA, USA).

Pancreatic islets were isolated as described in our previous work [17]. Briefly, 6-week-old male C57BL/6J mice (Slac, Shanghai, China) were used to isolate islets by collagenase V (Sigma-Aldrich) digestion, then the islets were cultured in RPMI-1640 medium with 10% FBS, 10000 units/mL penicillin, and 10000 μg/ mL streptomycin with 11.1 mM glucose. For islets experiments, islets were isolated from single animal and at least three parallel preparations were performed for each experiments. All animal care and experiments were permitted by Institutional Animal Care and Use Committees of Shanghai Institute of Materia Medica (No. 2013-10-WHY-12).

### Cell viability assay and Hoechst33342 staining

Palmitic acid (PA, Sigma-Aldrich, St. Louis, MO, USA)/Bovine serum albumin (BSA, Sigma-Aldrich) preparation was as previously described [13]. Firstly, MIN6 cells were seeded in 96-well plates and incubated with different concentration of compounds (1, 3, 10, 30, 100 μM, for nifedipine; 10, 30, 100, 200, 300 μM for diazoxide, respectively) in the presence/absence of 0.5 mM PA. Control cells were incubated with 0.5% BSA. After 48 h incubation, the cell viability was measured using 3-(4,5-dimethyl-2-thiazolyl)-2,5-diphenyl-2-H-tetrazolium bromide (MTT). Briefly, MIN6 cells were supplemented with MTT (0.5mg/ml) and incubated for 4 h. Then dimethyl sulfoxide (DMSO) solution was used to dissolve formazan crystals. Cell viability was calculated by the absorbance at 490 nm wavelength.

Hoechst33342 staining was performed as described in detail in our previous work [17]. Briefly, cells were planted into a six-well plate. After treatment with 0.5 mM PA and different concentration of compounds, the cells were fixed with 4% paraformaldehyde and then stained with Hoechst33342 (10 μg/ml) for 15 min. The apoptotic cells were observed and photographed under a fluorescence microscope (DP70, Olympus, Tokyo, Japan), and the apoptotic ratio in each group was calculated according to 10 random insights.

### Western blot analysis

After treatment as above described, MIN6 cells were lysed by RIPA buffer (Beyotime Bio, Shanghai, China). Then proteins were collected and separated by SDS–PAGE and transferred to membranes. After blocking, membranes were incubated with following primary antibodies with different dilutions: rabbit anti-phospho-eukaryotic translation initiation factor 2 (eIF2α, 1:1000, Cell Signaling, Beverly, MA, USA), rabbit anti-eIF2α (1:1000, Cell Signaling), mouse-anti-C/EBP-homologous protein (CHOP, F-168, 1:500, Santa Cruz, CA, USA), rabbit-anti-cleaved caspase-3 (Asp 175, 1:1000, Cell Signaling), rabbit-anti-phospho-c-Jun N-terminal protein kinase (JNK, 1:1000, Cell Signaling), rabbit-anti-JNK (1:1000, Cell Signaling), rabbit-anti-β-actin (1:5000, Sigma-Aldrich). Then horseradish peroxidase (HRP)-conjugated secondary antibodies (1:10000; Jackson Laboratories, PA, USA) and SuperSignal West Pico Chemiluminescence kit (Pierce, IL, USA) was used to visualize resulting immunocomplex. All blotting were performed at least three times, the optical density of each bands were analyzed by Image J software (NIH, MD, USA).

### Cell immunofluorescence

For cell immunofluorescence, MIN6 cells were grown on glass coverslips in a six-well plate and incubated with 0.5% BSA alone (control), PA/BSA or PA/BSA mixed with different compounds for 48 h. Then the cells were fixed in 4% paraformaldehyde at 4°C overnight. After fixation, the MIN6 cells were incubated with primary antibodies (mouse anti-CHOP, dilution 1:100; rabbit anti-insulin antibody, dilution 1:100, Cell Signaling) and followed by secondary antibodies (AlexaFluor 546-conjugated goat anti-mouse IgG and AlexaFluor 488-conjugated goat anti-rabbit IgG, dilution 1:400, Invitrogen, CA, USA). The nuclei of cells were stained by 4’-6-diamidino-2-phenylindole (DAPI), then coverslips were mounted and the image were taken by fluorescence microscopy (DP70, Olympus). For islet immunofluorescence, the islets were incubated with rabbit-anti-pancreatic/duodenal homeobox-1 (PDX1, dilution 1:100, Upstate, VA, USA) primary antibody and followed by Alexafluor 546-conjugated goat anti-rabbit IgG (Invitrogen). The PDX1-positive cell rate was calculated as PDX1-positive cell number divided by total islet cell number.

Terminal deoxynucleotidyl transferase dUTP nick end labeling (TUNEL) staining in islets were described in our previous work [18]. Briefly, after the islets were treated with PA in the presence/absence of different compounds, the apoptotic cells in islets were detected by an In Situ Cell Death Detection Kit (Roche, Mannheim, Germany) following its protocol. DAPI was used for visualization of nuclei. The TUNEL-positive cell number in islets was modified by islet area to assess cell apoptosis.

### Calcium mobilization assay

The calcium mobilization assay was described in our previous work [19]. Briefly, MIN6 Cells were cultured in 96-well plates for 24 h, and then the medium was replaced with 3 μM Fluo-4/AM (Invitrogen) and 2.5 mM probenecid (Sigma-Aldrich) in Hanks' balanced salt solution (HBSS, Beyotime). After 90 min incubation, the plates were washed with HBSS, and pre-incubated with different concentration of nifedipine and diazoxide for 10 min. Then 0.5 mM PA was used to stimulate calcium release and the fluorescence absorption of Fluo-4 was examined to calculate calcium release by Flexstation III (Molecular Devices, CA, USA).

### Glucose-stimulated insulin secretion

MIN6 cells or islets of approximately the same size (150 μm in diameter) were plated in 24-well plates (250,000 cells or 10 islets per well). Cells/islets were incubated with 0.5 mM PA in the presence/absence of different compound for 48 h. Then the medium was removed and pre-incubated by HBSS without glucose for 1 h at 37°C. Thereafter, the cells/islets were incubated in HBSS containing 5 mM glucose for 1 h and then in 25mM glucose for another 1 h. The supernatant was collected and the insulin concentration was measured by enzyme-linked immunosorbent assay (ELISA) (Millipore, MA, USA) according to its protocol.

### Data analysis

All data are expressed as mean ± SE. The comparison of different groups was assessed by two-tailed unpaired Student’s t-test or one-way-ANOVA followed by Dunnet’s post hoc test. P < 0.05 indicated statistically significant difference.

## Results

### Nifedipine and diazoxide protects MIN6 cells from PA-induced apoptosis

**Fig 1:**
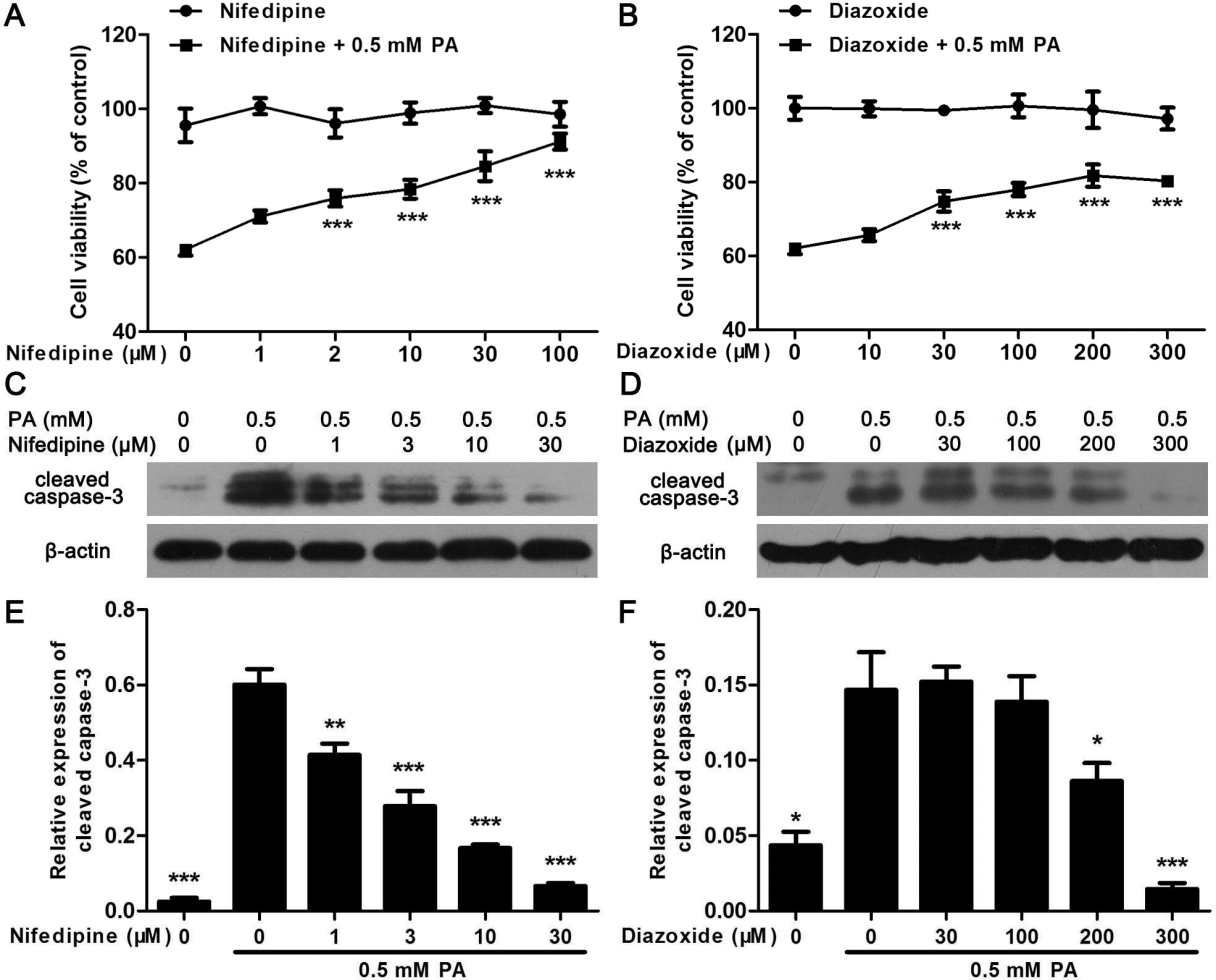
Nifedipine and diazoxide protects MIN6 cells from PA-induced apoptosis. (A) After 48 h incubation of nifedipine in the presence/absence of PA, the cell viability was measured by MTT assay. The cell viability was shown as inhibitory ratio (% of control), *** p<0.001 denote significant difference versus the PA-treated alone group, n = 6. (B) After 48 h incubation of diazoxide in the presence/absence of PA, the cell viability was measured by MTT assay. *** p<0.001 denote significant difference versus the PA-treated alone group, n = 6. (C) After the cells were treated with 0.5 mM PA in the presence/absence of nifedipine for 48 h, the expression of cleaved caspase-3 was detected by western blot. (D) After the cells were treated with 0.5 mM PA in the presence/absence of diazoxide for 48 h, the expression of cleaved caspase-3 was detected. (E)(F) Quantitative analysis of western blot. The optical density of each blot band was determined and adjusted by the optical density of β-actin. * p<0.05; ** p<0.01; *** p<0.001 denote significant difference versus the PA-treated alone group, n = 3.

There was less effect of both nifedipine and diazoxide on MIN6 cell viability after 48 h incubation (Fig 1A and 1B). However, 48 h treatment of 0.5 mM PA significantly reduced cell viability to approximately 60% compared to control cells (Fig 1A and 1B). Co-incubated with nifedipine and diazoxide dose-dependently inhibited PA-induced decreasing in cell viability (Fig 1A and 1B). Meanwhile, western blot analysis showed that the expression of cleaved caspase-3, an apoptotic marker, was obviously increased in PA-treated cells. Similarly, nifedipine reduced cleaved caspase-3 expression since 1 μM concentration (Fig 1C and 1E). Although not as effective as nifedipine, co-incubation of 200 μM diazoxide with PA still decreased cell apoptosis (Fig 1D and 1F).

**Fig 2:**
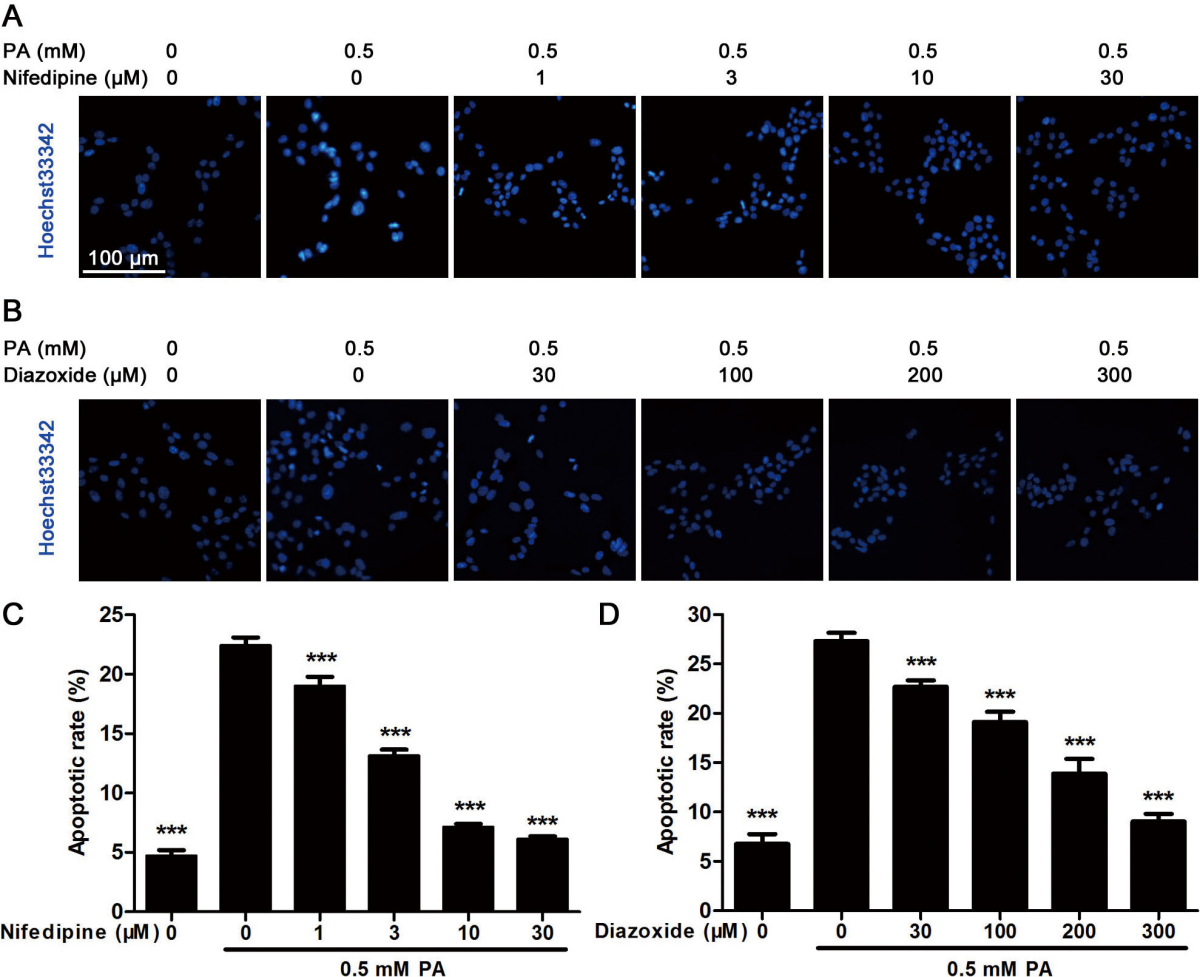
Hoechst33342 staining analysis in MIN6 cells. (A) After the cells were treated with 0.5 mM PA in the presence/absence of nifedipine at indicated concentrations for 48 h, Hoechst33342 staining was performed to detect apoptotic cells. Blue fluorescence indicated all nucleus, the lighter and shrinkage dots were apoptotic cells. Scale bar = 100 μm and referred to all panels. (B) After the cells were treated with 0.5 mM PA in the presence/absence of diazoxide for 48 h, Hoechst33342 staining was performed. (C)(D) The apoptotic rate was calculated as apoptotic cell number divided by total cell number. 10 random sights in each well were selected to count apoptosis, and the data from six duplicated wells were analyzed (n = 6). *** p<0.001 denote significant difference versus the PA-treated alone group.

To further evaluate the protective effect of nifedipine and diazoxide on MIN6 cells, Hoechst33342 staining was performed to visualize apoptotic cells. Nuclei of apoptotic cells would display a high condensed chromatin compared with normal cells after Hoechst staining. As shown in Fig 2A and 2B, 0.5 mM PA-treated cells showed brighter nuclei with nuclei shrinkage and highly condensed DNA. However, an obviously reduced apoptotic rate was observed in co-incubation of both nifedipine and diazoxide with PA compared to PA-treated alone group in a dose-dependent manner (Fig 2A–2D).

### Nifedipine and diazoxide attenuate PA-activated ER-stress in MIN6 cells

**Fig 3:**
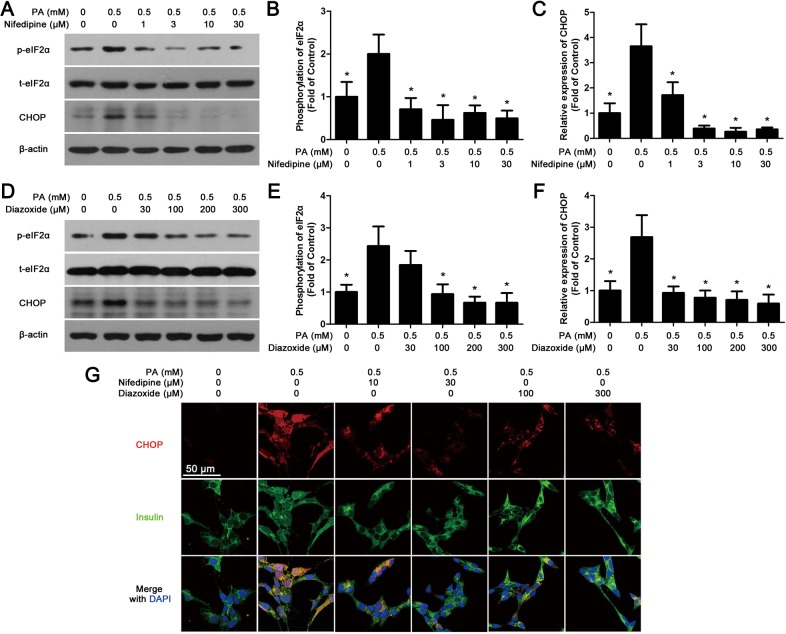
Nifedipine and diazoxide attenuate PA-activated ER-stress. (A) After MIN6 cells were co-treated with nifedipine and 0.5 mM PA for 48 h, the phosphorylation of eIF2α and the expression of CHOP were detected by western blot. β-actin was used for normalization. (B) The phosphorylation rate of eIF2α was calculated as optical density of phosphorylated-eIF2α (p-eIF2α) divide by total eIF2α (t-eIF2α). * p < 0.05 denote significant difference versus the PA-treated alone group, n = 3. (C) The expression of CHOP was calculated by optical density. * p<0.05 denote significant difference versus the PA-treated alone group, n = 3. (D) After MIN6 cells were co-treated with diazoxide and 0.5 mM PA for 48 h, the phosphorylation of eIF2α, the expression of CHOP were detected by western blot. (E) The phosphorylation rate of eIF2α was calculated by optical density. * p < 0.05 denote significant difference versus the PA-treated alone group, n = 3. (F) The expression of CHOP was calculated by optical density. * p < 0.05 denote significant difference versus the PA-treated alone group, n = 3. (G) After treatment, the cells were fixed and stained with CHOP and insulin antibodies. Red fluorescence indicated CHOP expression while green marked insulin. The nuclei were stained with DAPI dye. Scale bar = 50 μm and referred to all panels.

We than investigated the effects of nifedipine and diazoxide on the expressions of some ER-stress markers in PA-induced β-cell apoptosis. Fig 3 showed that after 48 h PA incubation, the expression of CHOP and phosphorylation rate of eIF2α were significantly increased in MIN6 cells. Nifedipine and diazoxide dose-dependently suppressed PA-induced phosphorylation of eIF2α. Meanwhile, both western blot (Fig 3A, 3C, 3D and 3F) and immunofluorescence (Fig 3G) experiments showed that the expression of CHOP in nifedipine or diazoxide co-treated with PA cells was obviously decreased in comparison with PA-treated alone group.

### PA-stimulated calcium release in MIN6 cells was inhibited by nifedipine and diazoxide under different conditions

**Fig 4:**
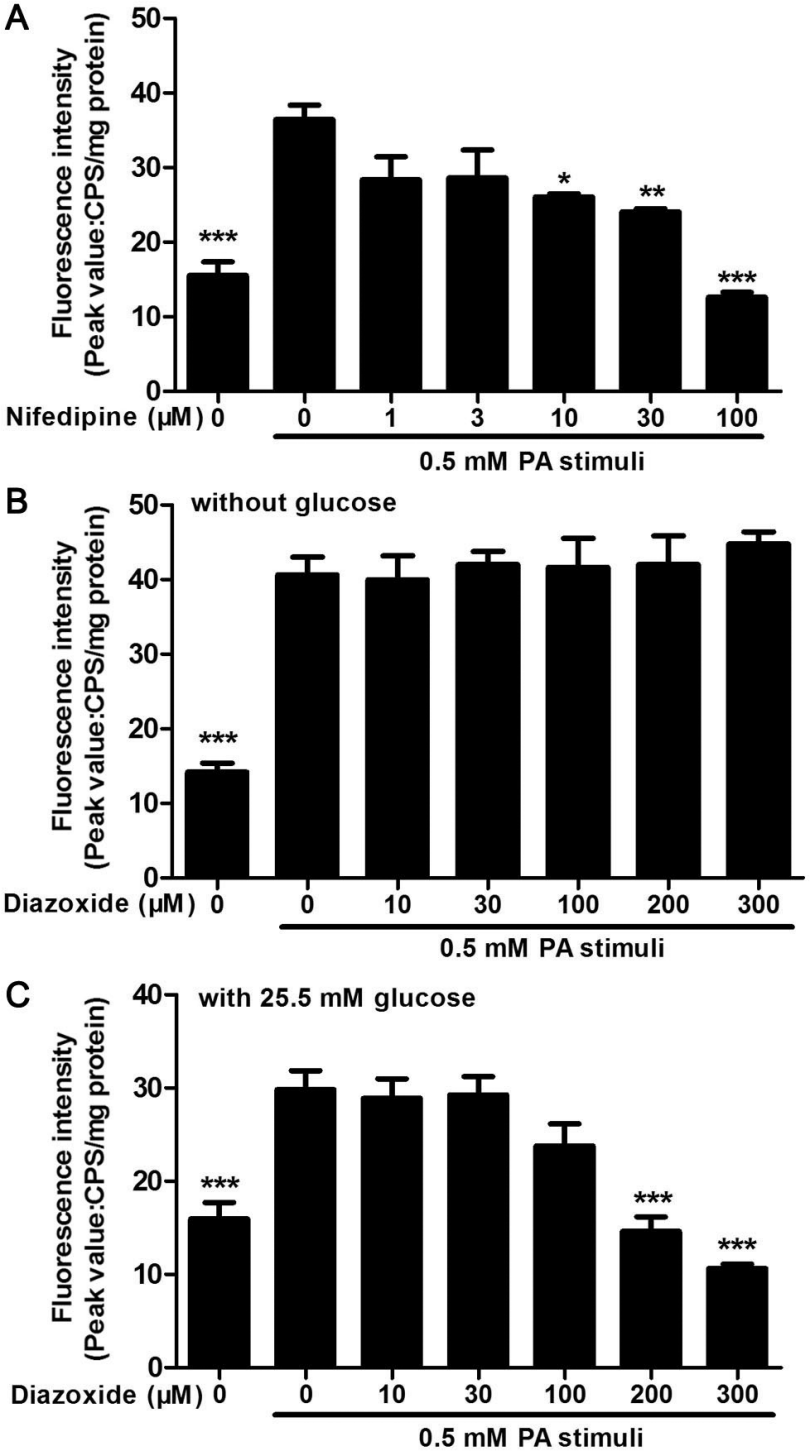
Nifedipine and diazoxide inhibited PA-stimulated Ca^2+^ release. (A) Pre-incubated of nifedipine dose-dependently inhibited 0.5 mM PA-stimulated Ca^**2+**^ release in MIN6 cells. (B) There was no significant change in PA-induced Ca^**2+**^ release between diazoxide co-treated and PA-treated alone group. The Ca^**2+**^ mobilization buffer did not contain glucose. (C) In the presence of 25.5 mM glucose, pre-incubated of diazoxide dose-dependently inhibited 0.5 mM PA-stimulated Ca^**2+**^ release in MIN6 cells. * p<0.05; ** p<0.01; *** p<0.001 denote significant difference versus the PA-treated alone group, n = 6.

To observe the effect of nifedipine and diazoxide on PA-stimulated Ca^2+^ release in MIN6 β-cells, the Fluo-4 dye was used to determine intracellular Ca^2+^ concentrations. It was found that nifedipine, a L-type Ca^2+^ channel blocker, produced a direct concentration dependent inhibition of PA-stimulated Ca^2+^ release in MIN6 cells (Fig 4A). And diazoxide, an ATP-sensitive K^+^ channel opener, could inhibit glucose-induced increasing of ATP concentration, thereby reducing membrane hyperpolarization and activation of voltage-dependent Ca^2+^ channel [20]. Thus, we also detected the effect of diazoxide on PA-stimulated Ca^2+^ release in MIN6 cells. However, diazoxide did not alter Ca^2+^ response to PA in MIN6 without glucose during monitoring time (Fig 4B). As diazoxide exerted protective effects on PA-impaired MIN6 cells, we then detected the PA-stimulated Ca^2+^ release in the presence of 25.5 mM glucose, which was the concentration of glucose used in common culture medium of MIN6 cells. It was found that diazoxide at 100 μM could slightly suppress PA-induced Ca^2+^ release in MIN6 cells, while 200 and 300 μM diazoxide significantly inhibited Ca^2+^ release in the presence of glucose.

### Nifedipine and diazoxide suppressed PA-induced phosphorylation of JNK

**Fig 5:**
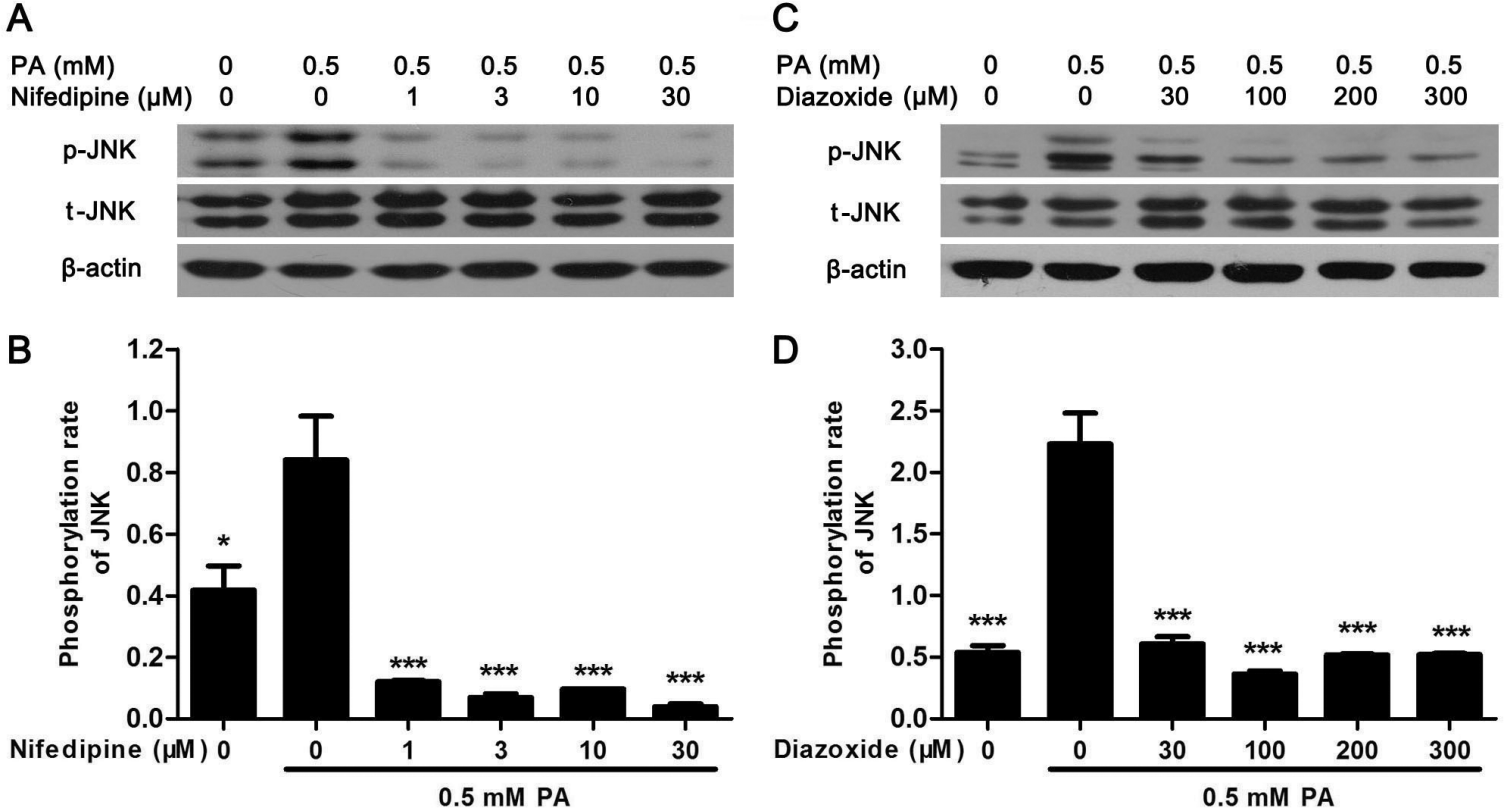
Nifedipine and diazoxide suppressed PA-induced phosphorylation of JNK. (A) After MIN6 cells were treated in 0.5 mM PA with/without different concentration of nifedipine for 48 h, the phosphorylation of JNK was detected by western blot. (B) The phosphorylation rate of JNK was calculated as optical density of phosphorylated-JNK (p-JNK) divide by total JNK (t-JNK). * p<0.05; *** p<0.001 denote significant difference versus the PA-treated alone group, n = 3. (C) After the cells were treated in 0.5 mM PA with/without different concentration of diazoxide, the phosphorylation of JNK was detected by western blot. (D) The phosphorylation rate of JNK was calculated. *** p<0.001 denote significant difference versus the PA-treated alone group, n = 3.

To further explore the signal pathway between calcium signal and apoptosis, the mitogen-activated protein kinase (MAPK) signal was examined. It was found that PA-activated JNK phosphorylation was significantly suppressed in MIN6 cell by nifedipine or diazoxide (Fig 5A–5C). However, no alteration was found in PA-activated extracellular signal-regulated kinase (ERK) and p38 MAPK (Data not shown), suggested that inhibition of JNK phosphorylation but not ERK or p38 MAPK might be involved in the cytoprotective effect of nifedipine and diazoxide on PA-impaired β-cells.

### Nifedipine and diazoxide improved GSIS function in PA-impaired pancreatic β-cells

**Fig 6:**
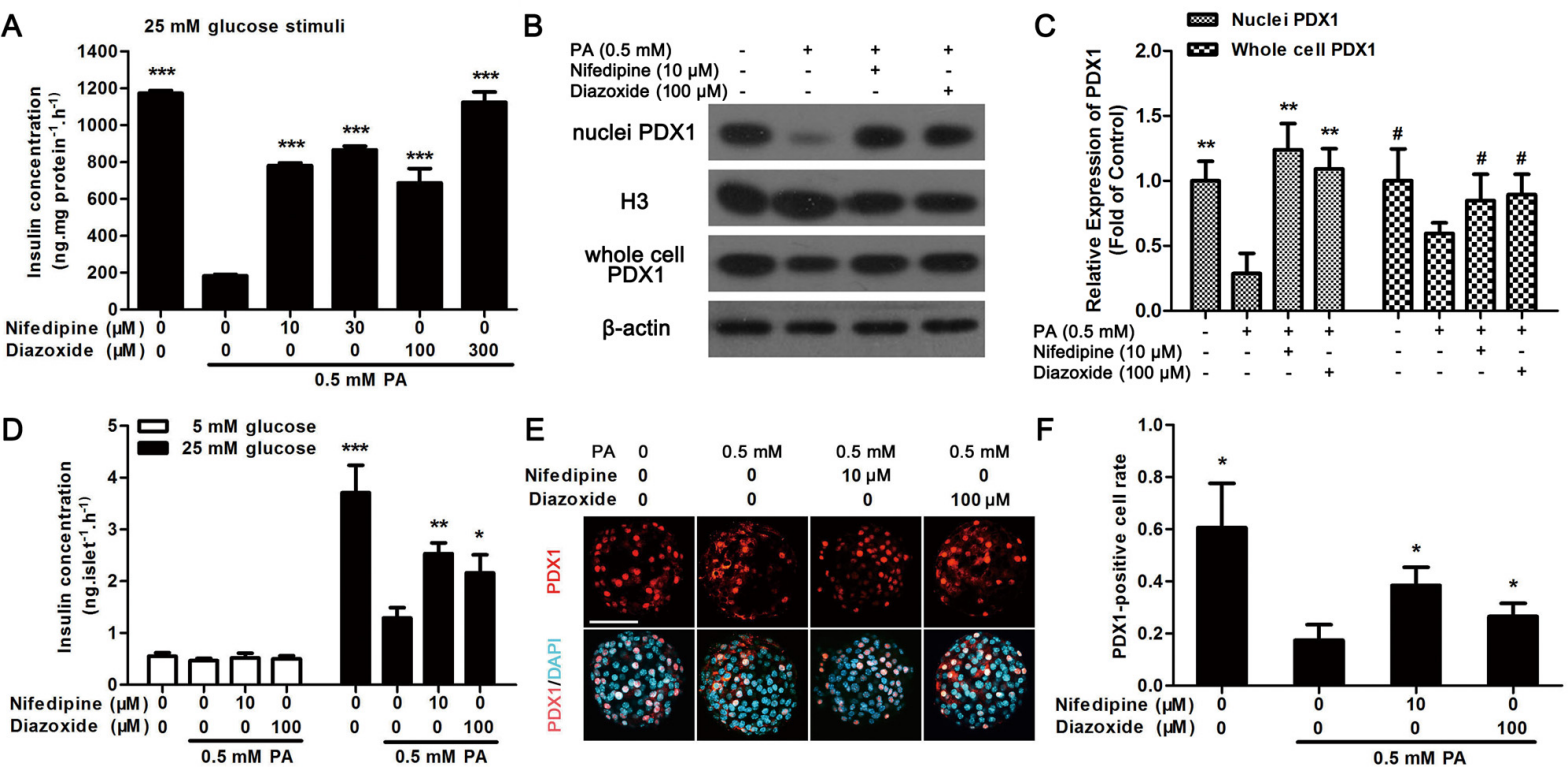
Nifedipine and diazoxide protected against PA-induced impairment of GSIS in pancreatic beta cells. (A) After MIN6 cells were treated with 0.5 mM PA in the presence/absence of nifedipine or diazoxide for 48 h, the GSIS induced by 25 mM glucose in MIN6 cells were determined. *** p<0.001 denote significant difference versus the PA-treated alone group, n = 6. (B) After MIN6 cells were treated with 0.5 mM PA in the presence/absence of nifedipine or diazoxide for 48 h, the expression of PDX1 in nuclei and whole cell was determined by western blot. (C) The expression of PDX1 was calculated by optical density. ** p<0.01 compared to PA-treated alone group in nuclei PDX1 detection; ## p<0.01 compared to PA-treated alone group in whole cell PDX1 detection, n = 3. (D) After cultured islets were treated with PA in the presence/absence of different compounds for 48 h, the GSIS induced by 5 mM and 25 mM glucose were detected. * p<0.05; ** p<0.01; *** p<0.001 denote significant difference versus the PA-treated alone group, n = 6. (E) Cultured islets were treated with PA in the presence/absence of different compounds for 48 h, then the PDX1 location was marked with red fluorescence, nuclei were dyed with DAPI to show blue fluorescence. Scale bar = 50 μm and referred to all panels. (F) The PDX1-positive cell rate was calculated as PDX1-postive nuclei number divided by total nuclei number in each islet. 10 islets were analyzed from six duplicated wells. * p<0.05 denote significant difference versus the PA-treated alone group, n = 6.

As nifedipine and diazoxide generated protective effect on PA-impaired β-cells, whether these two compounds could resist PA-induced impairment of insulin secretion in MIN6 β-cells and primary cultured islets was investigated. It was found that nifedipine at a concentration of 10 and 30 μM, diazoxide at 100 and 300 μM could partly restore PA-impaired GSIS in MIN6 cells (Fig 6A). We next detected the expression of PDX1 in nuclei and whole cell to investigate the translocation of PDX1. It was found that the nuclei PDX1 was significantly reduced after 48 h PA treatment in MIN6 cells (Fig 6B and 6C). However, both 10 μM nifedipine and 100 μM diazoxide increased the expression of nuclei PDX1 (Fig 6B and 6C). And PA-induced decreasing of PDX1 expression in whole cell was also significantly recovered by nifedipine and diazoxide (Fig 6B and 6C).

Moreover, as the results in MIN6 cells, after 48 h incubation, 10 μM nifedipine and 100 μM diazoxide could both increase 25 mM glucose-induced insulin secretion in PA-treated isolated islets in comparison with PA-treated alone islets (Fig 6D). And nifedipine or diazoxide co-treated with PA could slightly improve 5 mM glucose-stimulated insulin secretion, but this effect was not statistically significant (Fig 6D). Meanwhile, translocation of PDX1 to the nucleus was detected by immunofluorescence to further evaluate the protective effect of nifedipine or diazoxide on PA-treated islets. As shown in Fig 6E and 6F, PDX1 transported to nucleus was significantly reduced under PA-stimulation. Nonetheless, 10 μM nifedipine and 100 μM diazoxide partly restored the transport function of PDX1 to the nucleus (Fig 6E and 6F).

### Nifedipine and diazoxide decreased PA-induced apoptosis in cultured islets

**Fig 7:**
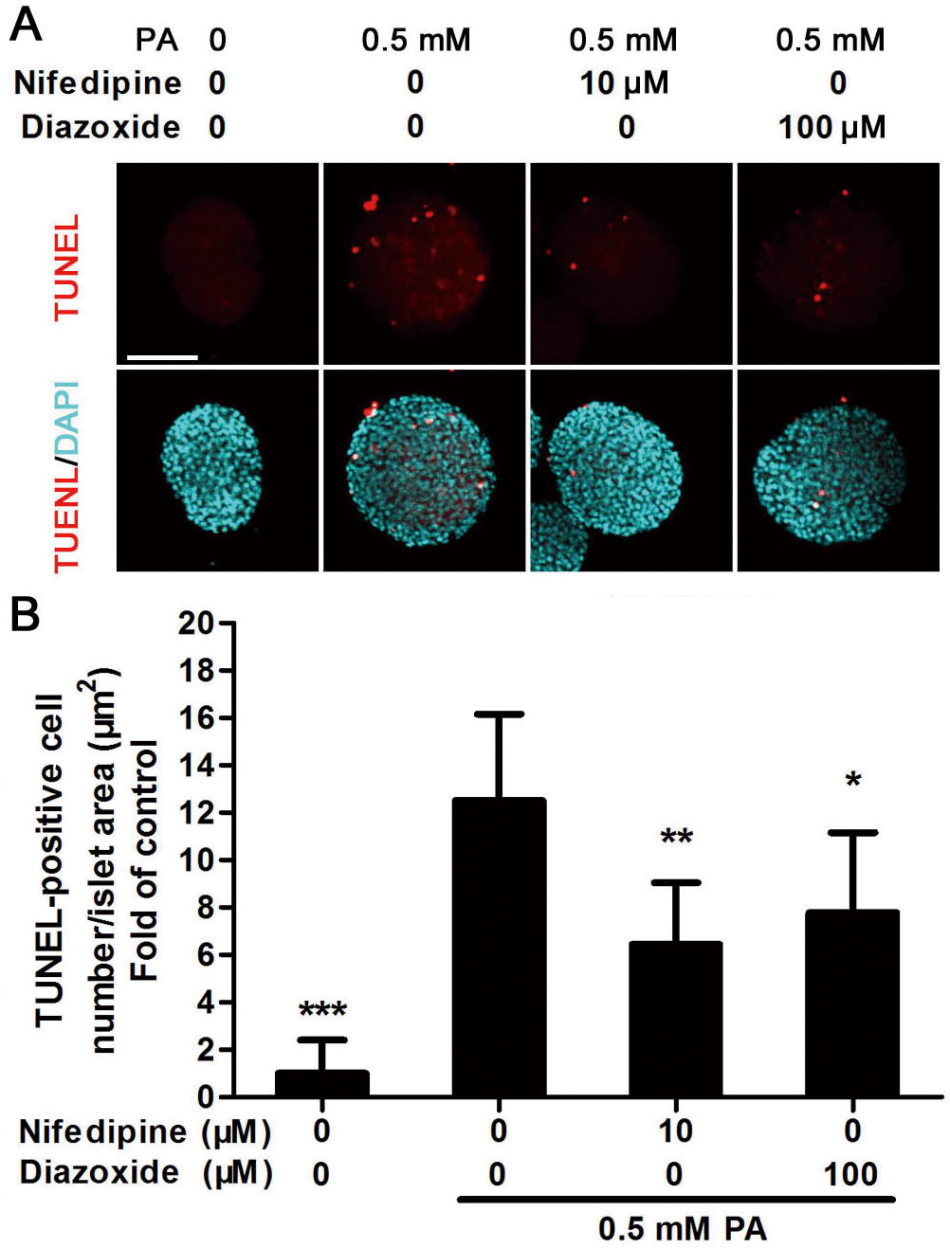
Nifedipine and diazoxide reduced cell apoptosis in PA-impaired islets. (A) Cultured islets were treated with PA in the presence/absence of different compounds for 48 h, then TUNEL staining was performed. Red fluorescence nuclei indicate apoptotic cells. Blue fluorescence showed all nuclei. Scale bar = 100 μm and referred to all panels. (B) Apoptotic rate was calculated as TUNEL-positive cell number modified by islet area. 10 islets were analyzed from six duplicated wells. * p<0.05; ** p<0.01 denote significant difference versus the PA-treated alone group, n = 6.

To further evaluate whether the two Ca^2+^ influx inhibitors, nifedipine and diazoxide, could reduce PA-induced apoptosis in primary cultured islets, TUNEL staining was used to indicate apoptosis by detecting DNA fragmentation. After the islets were treated with 0.5 mM PA for 48 h, the number of TUNEL-positive cells increased significantly compared to untreated islets (Fig 7A and 7B). As the experiments performed in MIN6 cells, nifedipine and diazoxide reduced PA-induced apoptosis in islets significantly (Fig 7A and 7B).

## Discussion

During the development of T2D, western diets rich in saturated fats increase plasma lipid levels, which leads to obesity and insulin resistance, thereby aggravating compensatory insulin secretion in pancreatic β-cells [21, 22]. Meanwhile, elevated FFAs could not be oxidized in mitochondria but shunted into the esterification, caused accumulation of long-chain acyl-CoA ester in cytoplasm, consequently induced lipotoxicity and directly affect β-cells [23]. Together factors could induce β-cell dysfunction and apoptosis in T2D. Thus, reducing lipotoxicity to improve pancreatic β-cell survival and function could provide benefit for improvement of T2D.

Chronic exposure to high levels of FFAs, especially saturated FFAs like PA, could activate Ca^2+^ signal and induce abnormal cytosolic Ca^2+^ homeostasis in β-cells, thereby causing stress reaction and cell apoptosis [11, 24]. Moreover, other stress factors, such as high level glucose and inflammatory cytokine induced apoptosis could be also associated with Ca^2+^ signal [25, 26]. Therefore, inhibition of Ca^2+^ release might provide benefit for β-cell protection. It was reported that using some Ca^2+^ influx blocker, including nifedipine and pituitary adenylate cyclase-activating polypeptide (PACAP), could reduce Ca^2+^ toxicity in islet β-cells [6, 25, 27]. Meanwhile, inhibition of GPR40-mediated PA-induced Ca^2+^ release could also reduce β-cell dysfunction [28]. Our previous study showed that using a small molecule GPR40 antagonist, DC260126, could reduce chronic FFAs-induced ER-stress and apoptosis in β-cells [13]. And inhibition of Ca^2+^ release by DC260126 also decreased hyperinsulinemia and protected β-cells in diabetic animals [18, 29]. These data were in according with results in the present study that using nifedipine or diazoxide, PA-induced β-cell apoptosis could be obviously reduced. However, Tan et al. reported that 10 μM diazoxide could not protect MIN6 cells from high glucose and FFAs-induced apoptosis [30], as other studies stated that 100 μM diazoxide generate protective effect on β-cells under Ca^2+^ signal-mediated impairment [15, 31]. The different dose used in these experiment might contribute to the diverse results. Interestingly, we confirmed that unlike nifedipine, diazoxide could not inhibit Ca^2+^ influx in MIN6 β-cells in the absence of glucose. Thus, the protective efficacy of diazoxide might be less than L-type Ca^2+^ channel blocker nifedipine. It might partially explain the controversial about the protective effect of diazoxide on lipotoxic β-cells. Anyhow, it could still inferred that the different inhibitory pattern of the two compounds, nifedipine and diazoxide, on Ca^2+^ influx was still associated with its protective effect on β-cells. On the contrary, some compounds, like etoposide [32], 2,3,7,8-Tetrachlorodibenzo-p-Dioxin (TCDD) [26] and human islet amyloid polypeptide [33] could activate Ca^2+^ influx, thereby causing β-cell death. Together results suggested that intervening Ca^2+^ release by compounds could provide benefits for β-cell protection.

It was reported that Ca^2+^ release from ER of β-cells could be observed in response to PA, thereafter this rapid depletion of ER Ca^2+^ could contribute to PA-induced ER-stress and apoptosis in β-cells [24, 34]. Meanwhile, sustained Ca^2+^ release increased demands on the ER for synthesis of insulin, subsequently increased unfolded protein response to initiate cell apoptosis [1]. During this process, one important ER-stress marker, CHOP induction in response to PA was also partially mediated by Ca^2+^ influx [10]. Another ER-stress marker, PRKR-like endoplasmic reticulum kinase (PERK) was also rapidly activated by PA in β-cells, thereby activating apoptotic proteins like caspase-3 [35]. We found that PA-activated CHOP expression and PERK-mediated eIF2α phosphorylation in ER-stress were significantly increased after chronic PA stimulation, which corroborated the results in our previous study [13]. Oppositely, using Ca^2+^ influx blockers nifedipine and diazoxide, PA-activated elevation of CHOP and eIF2α phosphorylation were obviously suppressed, suggested that inhibition of Ca^2+^ signal could be beneficial for reducing ER-stress and apoptosis in pancreatic β-cells. In addition, we also tested the effect of nifedipine and diazoxide on cytokines- and H_2_O_2_-treated MIN6 β-cell models. It was found that there was less effect of these two compounds on neither cytokines- nor H_2_O_2_-induced apoptosis in MIN6 cells (S1 Fig). Thus, the protective effect of nifedipine and diazoxide might be mainly ascribed to ER-stress but not oxidative stress or inflammation.

During the development of T2D, insulin resistance could increase insulin secretion to maintain normoglycemia, which termed β-cell compensation [36]. However, sustained elevation of insulin secretion finally causes β-cell exhaustion, subsequently induces apoptosis [36, 37]. Thus, inhibition of Ca^2+^ influx might not only suppress ER-stress to protect against β-cell apoptosis, but also reduce insulin secretion to decrease β-cell compensation. In the process of T2D, hyperglycemia and hyperlipidemia could activation JNK phosphorylation to augment Forkhead box protein O1 (FOXO1), which is a transcription factor to regulate insulin synthesis in β-cells [38]. FOXO1 could promote PDX1 translocation into nucleus, consequently augment insulin secretion [38, 39]. On the contrary, chronic exposure to PA inhibits insulin gene transcription via altering PDX1 nuclear localization [39]. It was showed that nifedipine and diazoxide could both partially increase PA-impaired PDX1 expression and translocation to nuclei, thereby restoring GSIS of β-cells. Moreover, JNK activation could also directly induced ER-stress and apoptosis in β-cells, whereas suppression of JNK phosphorylation by specific inhibitor, such as SP600125, could improve β-cell survival both in vitro and in vivo [38, 40]. In according with these results, we found that PA-induced JNK activation could be approximately abolished by nifedipine and diazoxide. It was reported that Ca^2+^ could activate JNK via calmodulin-dependent protein kinase II (CaMK II) [41, 42]. And JNK phosphorylation could directly promote ER-stress and β-cell apoptosis [11, 38]. Therefore, inhibition of JNK phosphorylation might be associated with the protective effect of the two Ca^2+^ influx blockers on pancreatic β-cells. However, the detailed relationship between Ca^2+^ influx and lipotoxicity in pancreatic β-cells still need further investigation.

## Conclusions

It was found that both nifedipine and diazoxide could inhibit PA-stimulated Ca^2+^ release, thereby reducing PA-mediated lipotoxicity in pancreatic β-cells. And inhibition of Ca^2+^ release also suppressed chronic sustained insulin secretion to reduce β-cell decompensation. These present data provided one possibility that inhibition of chronic FFAs-stimulated insulin secretion by regulation Ca^2+^ release might provide benefit for β-cell protection in T2D.

## Supporting Information

S1 Fig Nifedipine and diazoxide had less effect on H_2_O_2_ and cytokine-treated MIN6 cells. (DOCX)
