# Supplementary material for: Inhibition of Calcium Influx Reduces Dysfunction and Apoptosis in Lipotoxic Pancreatic β-Cells via Regulation of Endoplasmic Reticulum Stress
Source: PLoS One. 2015 Jul 6;10(7):e0132411. doi: 10.1371/journal.pone.0132411 (PMC4492560; doi:10.1371/journal.pone.0132411)

**S1 Fig. Nifedipine and diazoxide had less effect on H_2_O_2_ and cytokine-treated MIN6 cells.** (A) Different concentration of nifedipine were pre-incubated with MIN6 cells for 2 h. Then the cells were stimulated for another 2 h by 0.5 mM H_2_O_2_ in the presence of compound. After stimulation, the cell viability was measured by MTT assay. ∗∗∗ p<0.001 denote significant difference versus the H_2_O_2_-treated alone group, n=6. (B) Different concentration of diazoxide were pre-incubated with MIN6 cells for 2 h. Then the cells were stimulated for another 2 h by 0.5 mM H_2_O_2_ in the presence of compound. After stimulation, the cell viability was measured by MTT assay. ∗∗∗ p<0.001 denote significant difference versus the H_2_O_2_-treated alone group, n=6. (C) After 48 h incubation of different dose of nifedipine in the presence/absence of 5 ng/ml IFN-γ, 5 ng/ml IL-1β and 30 ng/ml TNF-α, the cell viability was measured by MTT assay. ∗∗∗ p<0.001 denote significant difference versus the cytokine-treated alone group, n=6. (D) After 48 h incubation of different dose of diazoxide in the presence/absence of 5 ng/ml IFN-γ, 5 ng/ml IL-1β and 30 ng/ml TNF-α, the cell viability was measured by MTT assay. ∗∗∗ p<0.001 denote significant difference versus the cytokine-treated alone group, n=6.


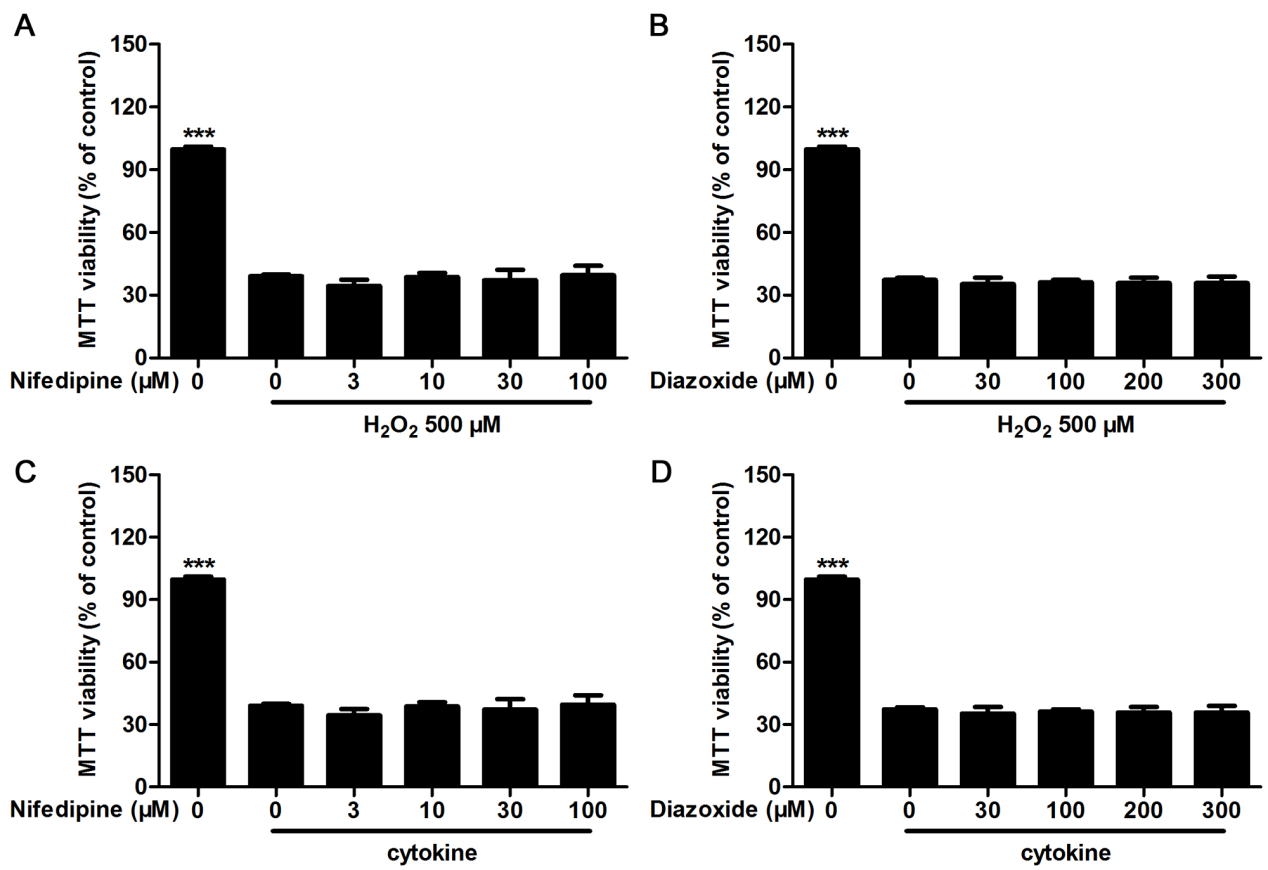

Supplement: S1 Fig — (DOCX) [file pone.0132411.s001.docx]
